# Dysfunction and Dysconnection in Cortical–Striatal Networks during Sustained Attention: Genetic Risk for Schizophrenia or Bipolar Disorder and its Impact on Brain Network Function

**DOI:** 10.3389/fpsyt.2014.00050

**Published:** 2014-05-09

**Authors:** Vaibhav A. Diwadkar, Neil Bakshi, Gita Gupta, Patrick Pruitt, Richard White, Simon B. Eickhoff

**Affiliations:** ^1^Department of Psychiatry and Behavioral Neurosciences, Wayne State University, Detroit, MI, USA; ^2^Institute of Clinical Neuroscience and Medical Psychology, Heinrich-Heine University Düsseldorf, Düsseldorf, Germany; ^3^Institute of Neuroscience and Medicine (INM-1), Research Center Jülich, Jülich, Germany

**Keywords:** attention, brain networks, schizophrenia, bipolar disorder, dynamic causal modeling abstract

## Abstract

Abnormalities in the brain’s attention network may represent early identifiable neurobiological impairments in individuals at increased risk for schizophrenia or bipolar disorder. Here, we provide evidence of dysfunctional regional and network function in adolescents at higher genetic risk for schizophrenia or bipolar disorder [henceforth higher risk (HGR)]. During fMRI, participants engaged in a sustained attention task with variable demands. The task alternated between attention (120 s), visual control (passive viewing; 120 s), and rest (20 s) epochs. Low and high demand attention conditions were created using the rapid presentation of two- or three-digit numbers. Subjects were required to detect repeated presentation of numbers. We demonstrate that the recruitment of cortical and striatal regions are disordered in HGR: relative to typical controls (TC), HGR showed lower recruitment of the dorsal prefrontal cortex, but higher recruitment of the superior parietal cortex. This imbalance was more dramatic in the basal ganglia. There, a group by task demand interaction was observed, such that increased attention demand led to increased engagement in TC, but disengagement in HGR. These activation studies were complemented by network analyses using dynamic causal modeling. Competing model architectures were assessed across a network of cortical–striatal regions, distinguished at a second level using random-effects Bayesian model selection. In the winning architecture, HGR were characterized by significant reductions in coupling across both frontal–striatal and frontal–parietal pathways. The effective connectivity analyses indicate emergent network dysconnection, consistent with findings in patients with schizophrenia. Emergent patterns of regional dysfunction and dysconnection in cortical–striatal pathways may provide functional biological signatures in the adolescent risk-state for psychiatric illness.

## Introduction

Sustained attention or the ability to remain consistently focused on an ongoing task is one of the most basic of cognitive domains (1, 2) and serves as a fundamental process underlying mechanisms of memory and control (3). Attention competence in childhood and adolescence increases through emergence of functional integration within cortical–striatal circuits. The engagement of frontal regions has been documented in children as young as 4–6 years of age (4) and the maturation of the circuit (including the basal ganglia and the parietal lobe) extends through adolescence (3, 5). This multi-node attention network (6) includes executive regions of the frontal lobe (the dorsal prefrontal cortex and the dorsal anterior cingulate), regions such as the basal ganglia (including the caudate and the putamen) that presumably play central roles in relaying information between and linking signals across brain networks (7, 8), and the parietal lobe that is essential for mechanisms of spatial orientation (9). The ascent of attention competence in adolescence corresponds with linear progression in the development of and anatomical connectivity between these key brain structures in the attention network (10, 11).

Deficits in sustained attention deficits are widely implicated in several psychiatric disorders that are adolescent onset or the origins of which lie in adolescence. These include not only core attention-related disorders such as attention deficit hyperactivity disorder (6, 12), but also bipolar disorder (13) and schizophrenia (14). The evidence regarding schizophrenia and bipolar disorder is compelling as studies now suggest attention deficits serve as a prelude in adolescence to the emergence of these late adolescent or adult-onset phenotypes. In this framework, adolescents with known risk-factors for psychiatric illness may present with neuropsychological deficits, which in turn are expressions of emergent dysfunction in critical brain networks (15). Adolescent children of parents with psychiatric diagnoses (mood disorders or schizophrenia) are an important risk group in whom familial risk may impact the integrity of function in attention networks, in turn decreasing the integrity of attention-related processing and subsequently leading to an increase in expressed attention deficits. In fact, adolescent children of parents with major depressive disorder, bipolar disorder, or schizophrenia all show deficits in neuropsychological tasks of attention including continuous performance tasks (CPT) (14, 16, 17) and other tasks with significant attention components (17). These groups are at significantly higher risk (HGR) for the emergence of psychiatric disorders (18–21). Consequently, a better understanding of the neurobiological impairments of attentional networks may provide important insight and potential biomarkers for the emergence of these disorders.

However, understanding of these biological bases remains obscure. Volumetric studies imply cortical–striatal reductions in brain structure (22, 23) that may be associated with impaired attention function (24, 25). However, the relationship between brain structure and function (as measured with structural and functional MRI, respectively) is not straightforward (26). This limits insight into disordered brain function in adolescence and its implication for psychiatric illness. In turn, understanding disordered effective connectivity between brain regions using causal modeling of brain network interactions assumes particular importance for understanding dysregulated networks in psychiatric disorders (27–29).

Effective connectivity mediates the integration of information between brain regions and refers to the “the influence that one neural system exerts over another, either at a synaptic (i.e., synaptic efficacy) or population level” (27, 30). Assessing brain activations and effective connectivity respectively permit exploration of relative specialization and functional integration of information in the brain (27). The temporal properties of the BOLD response, and the relationship of this to biophysical forward models of the neuronal response (31) permit the modeling of and inference on parameters of effective connectivity estimated from fMRI (32). While different methods for analyzing effective connectivity exist, dynamic causal modeling (DCM) is the currently best evaluated and most widely used approach toward this endeavor (32–35).

Our aims in this investigation were twofold. First, we assessed differences in regional responses across the extended cortical–striatal attention network (36, 37) including frontal, striatal, and parietal cortices. These differences in part constitute differences in the regional specialization of function between groups. Next, using DCM (33), we investigated differences between cortical–striatal network interactions using a competitive network identification framework based on Bayesian model selection (BMS) (38) and comparisons of Bayesian parameter averages (34, 39). fMRI data were collected in children and adolescents (8 years ≤ Age < 20 years) with a family history of psychiatric illness (bipolar disorder or schizophrenia) (henceforth HGR) and controls free of such history to the second degree [henceforth typical controls (TC)]. During the fMRI task, extended attention blocks (120 s) were employed using a variant of the well-established CPT (identical pairs version, CPT-IP) (40) in which subjects must monitor rapidly presented stimuli (in the current context numbers were used) and indicate repetitions in the sequence.

## Materials and Methods

### Subjects

A total of 46 children and adolescents provided informed consent or assent for the fMRI studies approved by the institutional review board at Wayne State University. Of these 46, 24 were TC, with no family history of schizophrenia or mood disorder to the second degree and remaining 22 had a parent with schizophrenia or bipolar disorder and hence at HGR. Subjects were recruited from the greater Detroit area through advertisements and through in patient services at Wayne State University School of Medicine. Screening questionnaires administered using both telephone and personal interviews were used for both rule-outs and to ascertain if subjects had a history of psychotic illness in first-degree relatives. Diagnoses for parents of HGR were reached using the Structured Clinical Interview for DSM-IV schizophrenia (41). Subjects younger than 15 years were clinically characterized using the Schedule for Affective Disorders and Schizophrenia-Child Version (K-SADS) (42); those aged 15 years or above were assessed using the SCID. Table 1 provides information on subject demographics and characteristics.

**Table 1:** **Demographic information for the investigate sample is shown**.

	Mean age (SD)	Full scale IQ (SD)
Typical controls (TC, *n* = 24)	15.4 (2.7)	93.1 (15.9)
High genetic risk (HGR, *n* = 22)	14.1 (3.1)	94.2 (14.5)

*HGR were healthy apart from the following co-morbidities: separation anxiety (*n* = 1), attention deficit hyperactivity disorder (*n* = 3), and social phobia (*n* = 1). TC by definition were healthy and free of diagnosis*.

### fMRI

Functional data were acquired using a full body Bruker MedSpec 4.0 T system running the Siemens Syngo console. Gradient echo planar images (EPI) were collected using an eight-channel head coil (TR = 2000 ms; TE = 30 ms; matrix size = 64 × 64; field of view (FOV) = 240 mm; voxel size = 3.75 mm × 3.75 mm × 4 mm). Images were axially acquired in 24 continuous 4 mm slices positioned parallel to the anterior commissure/posterior commissure (AC–PC) line.

### Task

During fMRI, all subjects performed a modified version of a CPT (Identical Pairs version) previously employed in studying illnesses including schizophrenia and bipolar disorder, and children and adolescents at risk for psychiatric illness (14, 16, 40). Numbers were presented in rapid sequence (50 ms, 250 ms SOA in each condition) and subjects were required to detect the repeated presentation of a number. Attention demands of the task were maintained by manipulating figure-ground contrast (white characters, RGB: 255, 255, 255; Off-white background, RGB: 225, 225, 225) in order to preempt attention gain under maximal contrast (43). Attention load was manipulated across epochs utilizing sequences of two-digit numbers (“low” load) or three-digit numbers (“high” load), motivated by evidence suggesting that access to numerosity though rapid (44) interacts with attention systems in the frontal, striatal, and parietal regions (45–47). A goal of manipulating load was to investigate separable load-related effects on region-specific interactions in each experimental group, particularly as parametric variations in load have proven useful in assessing differential regional specialization in risk and disease (48, 49).

**Figure 1:**
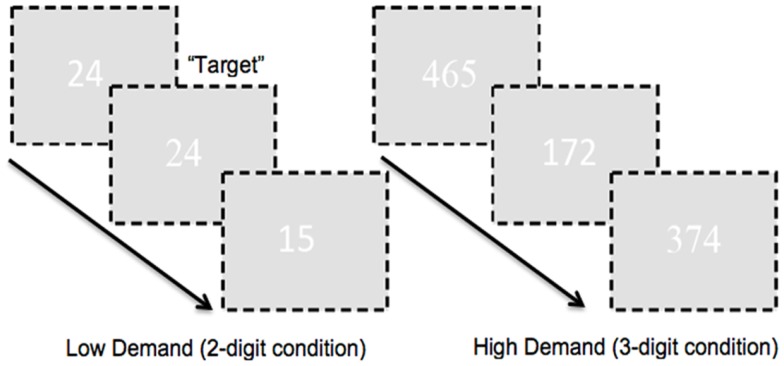
**A schematic of the employed CPT-IP task is shown**. The task alternated between two- and three-digit extended epochs and subjects were required to detect repeated presentation of numbers. As seen attention demand was modulated by manipulating figure-ground contrast. The numbers in the figure were RGB: 255, 255, 255; the ground was RGB: 225, 225, 225 in order to minimize contrast. In addition, font between successive numbers alternated (arial, times new roman). This ensured a baseline level of “flicker” between successive presentations, pre-empting target detection based on visual cues such as the absence of flicker.

To ensure large effect sizes of continuous or sustained attention, we used very long blocks of 120 s (therefore in removing low frequency drifts and fluctuations in subsequent analyses, we used a lenient high pass filter to preserve attention-related responses in the fMRI signals further noted in the fMRI analyses section below). Target frequency during the 120 s experimental epochs was 25%. In addition to experimental epochs, we also employed corresponding two- or three-digit control epochs (for each corresponding level of demand). During these epochs subjects passively observed two- or three-digit strings (“00” or “11”; “000” or “111”). Pure rest epochs (20 s) were also interspersed throughout the experimental run. Subjects signaled responses by button press on a standard response box. A schematic of the task is presented in Figure 1.

### fMRI processing (activation analyses)

Data were processed with Statistical Parametric Mapping (SPM8). Realignment was performed to correct for head motion artifact during the scan. Realigned images were normalized to the Montreal Neurological Institute (MNI) EPI template and voxels resliced (2 mm × 2 mm × 2 mm). Normalized images were smoothed using an 8 mm FWHM Gaussian kernel. Images where estimated motion exceeded 4 mm were discarded from the analyses (<1% of all images).

In the first (within-subject) level analyses, rest, control, and attention epochs were modeled with boxcar stimulus functions that were convolved with a canonical hemodynamic response function to form regressors. Serial correlations were modeled with an auto-regressive process and low frequency fluctuations were removed with a high pass filter (using a discrete cosine set covering frequencies of 1/256 s or lower). Note that we did not model phasic or event related responses to targets. This was because we were primarily interested in the responses associated with sustained attention.

First level contrasts for each level of demand relative to the corresponding control condition (Attention > Control) were computed for each individual subject. That is, we contrasted the beta-estimates for the low-attention condition with those for passively viewing two-digit strings and those for the high attention condition with those for passively viewing three-digit strings. This was performed to identify responses to attention-related (as opposed simply to visual) processing. First level maps were submitted to second level analyses of covariance with Group (HGR, TC) as the independent factor, demand (two-digit vs. three-digit) as non-independent factor, and age, gender, and task performance (assessed with *d*′)(50) as covariates. Clusters of activation (*p* < 0.05, cluster level corrected for multiple comparisons) (51) were employed to identify significant brain regions for each of the effects.

### fMRI processing (linear DCM analyses)

More formal coverage of DCM can be found elsewhere (33, 52, 53). Briefly, DCM allows the interpretation of causal interaction between hidden state variables (32). The brain is viewed as a bi-linear input (experimental conditions) – output (fMRI measured hemodynamic response) system. Changes in the neural responses are modeled using the following state differential equation:

$$\frac{dx}{dt}=\left(A+\sum_{j=1}^{m}u_{j}B^{\left(j\right)}\right)x+Cu.$$

where, *A* represents task-independent endogenous coupling between regions, *B*^(^*^j^*^)^ represents putative modulation of endogenous connections by experimental manipulations (e.g., Attention, *u_j_*), and *C* represents sensorimotor driving inputs on (typically) unimodal cortical regions.

A goal of DCM is to identify model(s) with the highest evidence given the observed fMRI data by testing competing hypotheses on a model space (54). Therefore, assessment of effective connectivity using DCM requires evaluation and comparison of neurobiologically plausible competing models, each representing hypotheses on the connective-architecture of the investigated neural system.

**Figure 2:**
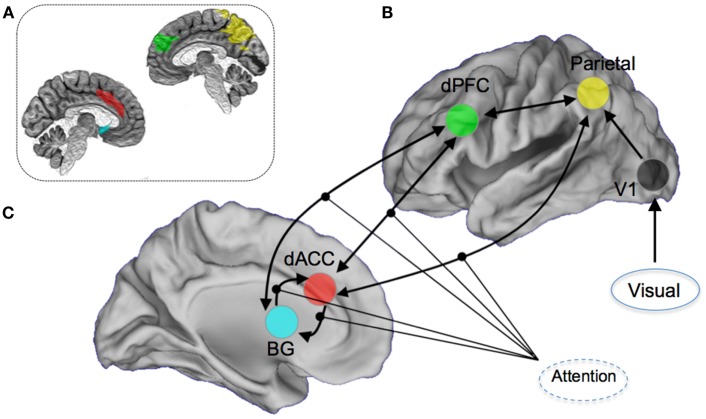
**Overview of the DCM model space**. Seventy-two competing models were constructed by permuting the modulation of attention on **(A)** dorsal anterior cingulate (dACC) efferent pathways to the Basal Ganglia (BG), the dorsal prefrontal cortex (dPFC), and the parietal lobe, **(B)** dPFC and BG, and **(C)** BG and the dACC. Visual inputs to the system were modeled through the primary visual cortex. For the dACC–BG pathway, the bilateral endogenous connection itself was permuted. The inset image provides a schematic depiction (on a mid-sagittal slices) of the anatomical definitions used in summarizing regional activity for the DCM modeling. The color-coding of the regions of interest is approximately maintained.

The *a priori* attention network of interest included regions both within the executive network (dACC, dPFC, and caudate nucleus) and sensory and spatial attention-related regions (parietal cortex and visual cortex) (6, 36, 37, 55). The particular focus of the modeling space (competing hypothesis) was the role of the dorsal anterior cingulate cortex and the contextual modulation of its efferent connections to other regions of the attention network. This approach was motivated in large part by the significant role played by the dACC in cognitive and resource control as it relates to attention and conflict (56), and its particular place in the control-related hierarchy of the forebrain (57, 58). Notably, disordered cognitive control has emerged as a general framework for understanding the schizophrenia and bipolar diathesis which the at-risk participants in our sample fall under (29, 59–61). DCM was implemented using DCM8 in SPM8. An *a priori* network of nodes was derived using regions of interests in stereotactic space (62). Within each structurally defined node of this network, we summarized regional activity on a subject-specific basis employing the principal eigenvariate of voxels within a 5 mm radius of the peak. Figure 2 shows the resulting architecture.

### Model estimation

Prior to modeling, time series were extracted from each region of interest (ROI) according to established procedures (63, 64) using spheres (5 mm radius) centered on the peak of the “effects of interest” *F*-contrast (*p*_FWE_ < 0.05, adjusted for “effects of no interest”). Each of the 72 models was estimated across subjects. To determine the most likely generative model, a random-effects (RFX) BMS procedure was applied. In particular, we used the variational Bayes method to estimate posterior probabilities of competing models. Bayesian parameter averages of coupling estimates (with a focus on modulatory coupling) were analyzed to determine potential differences in modulation as a function of attention and statistical significance was assessed using Bonferroni correction (*p* < 0.05)(32, 34).

## Results

### Behavioral results

Behavioral performance, which is the sensitivity to distinguish targets from distracters was assessed using *d*′ (65), an established metric in Signal Detection theory (50, 66). The metric incorporates the hit-rate (e.g., the rate of responding “same” to successively presented stimuli in the same valence category) and the false alarm rate (e.g., the rate of responding “different” to successively presented stimuli in difference valence categories), and is based on the difference between the inverse function of the cumulative Gaussian distribution applied to each, with a higher *d*′ reflecting greater sensitivity to the task.

**Figure 3:**
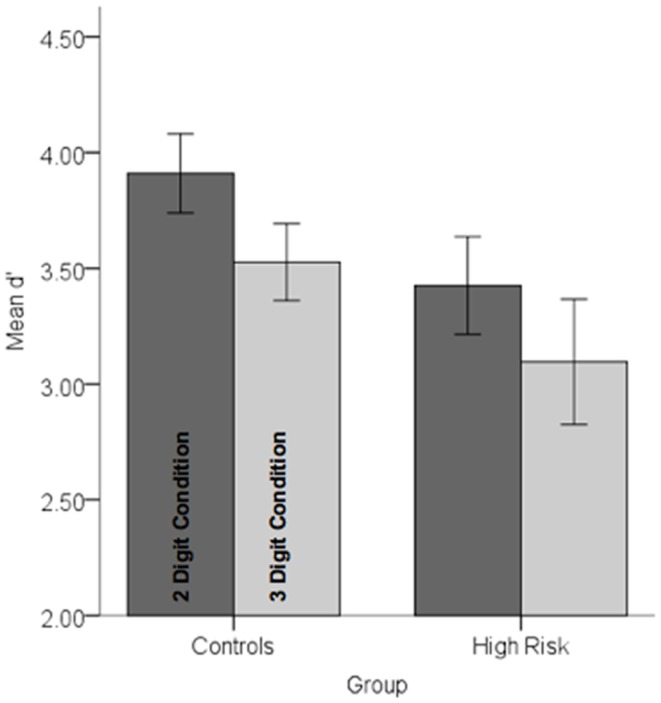
**Performance data (*d*′) in each of the controls and high-risk groups are depicted for each of the conditions**. Note that there was a marginally significant decrement in performance in the high-risk group and a highly significant effect of load (see text for statistical details). Error bars are ±SEM.

Behavioral data were analyzed in a repeated measures analysis of variance with Group (HGR vs. TC) as between subjects’ factor and attention demand (two-digit vs. three-digit) as within-subjects factor. The main effect of load was significant indicating that attention load reduced the sensitivity of observers, *F*_1,45_ = 11.67, *p* < 0.001, MSe = 0.25. A main effect of group was marginally significant, *F*_1,45_ = 2.86, *p* < 0.05, one-tailed, MSe = 1.71 suggesting that subjects with a family history of psychiatric illness were marginally less sensitive than controls. No other effects reached significance. Figure 3 depicts performance data across conditions and groups.

### Activation analyses with fMRI (differences in regional specialization of function)

A significant main effect of Group (HGR ≠ TC) and Group × Demand interaction was investigated in the constituent regions across the network of interest. Significant clusters under the main effect were observed in both the dorsal prefrontal cortex and the parietal lobe (*p* < 0.05, cluster level) and significant clusters under the interaction term were observed in the basal ganglia. Directionality (HGR ≠ TC) of the statistical effects and the interaction terms were inferred based on estimates of the modeled responses extracted under the overall peak within the cluster of significance.

**Figure 4:**
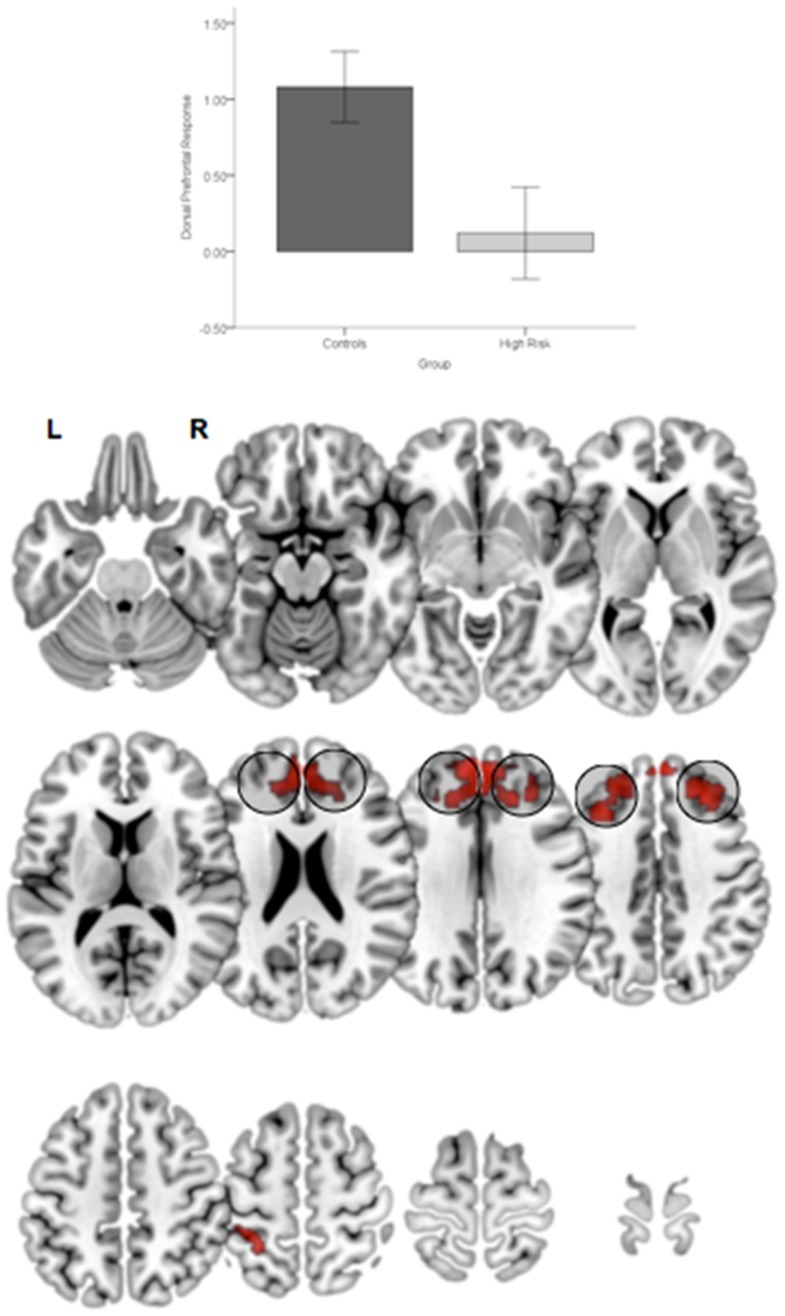
**Significant (*p* < 0.05, cluster level) bilateral clusters in the dlPFC and dmPFC (insets) under the overall main effect of group in the activation analyses represent significant hypo-engagement of the structure in HGR compared to TC (graph of parameter estimates from the peak in the dlPFC)**. The clusters are rendered on an ascending mosaic of axial surfaces. These results imply significant hypo-engagement of the prefrontal cortex in HGR during sustained attention.

**Figure 5:**
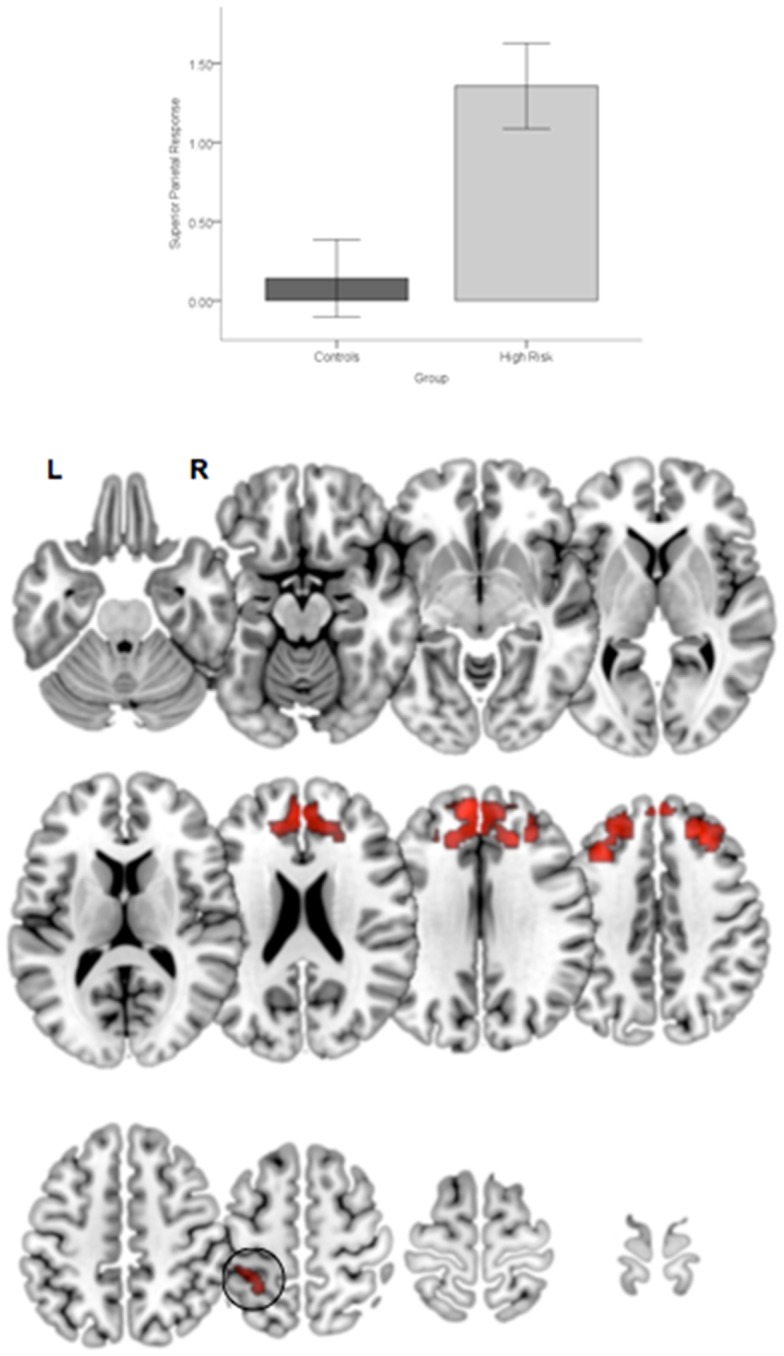
**Significant clusters (*p* < 0.05, cluster level) under the overall main effect of group in the activation analyses represent significant hyper-engagement of the parietal cortex (inset) in HGR compared to TC (see graph of parameter estimates from the peak in parietal cortex)**. The clusters are rendered on an ascending mosaic of axial surfaces. These results imply that HGR may inappropriately hyper-activate the parietal cortex during attention, suggesting an imbalance in the relative specialization of function underlying sustained attention.

First, relative to TC, HGR subjects evinced reduced engagement of the dorsal prefrontal cortex irrespective of the degree of attention demand. Figure 4 depicts significant clusters rendered on lateral and medial surfaces of the cortex. By comparison, HGR evinced increased engagement of the parietal cortex irrespective of the degree of attention demand (Figure 5).

**Figure 6:**
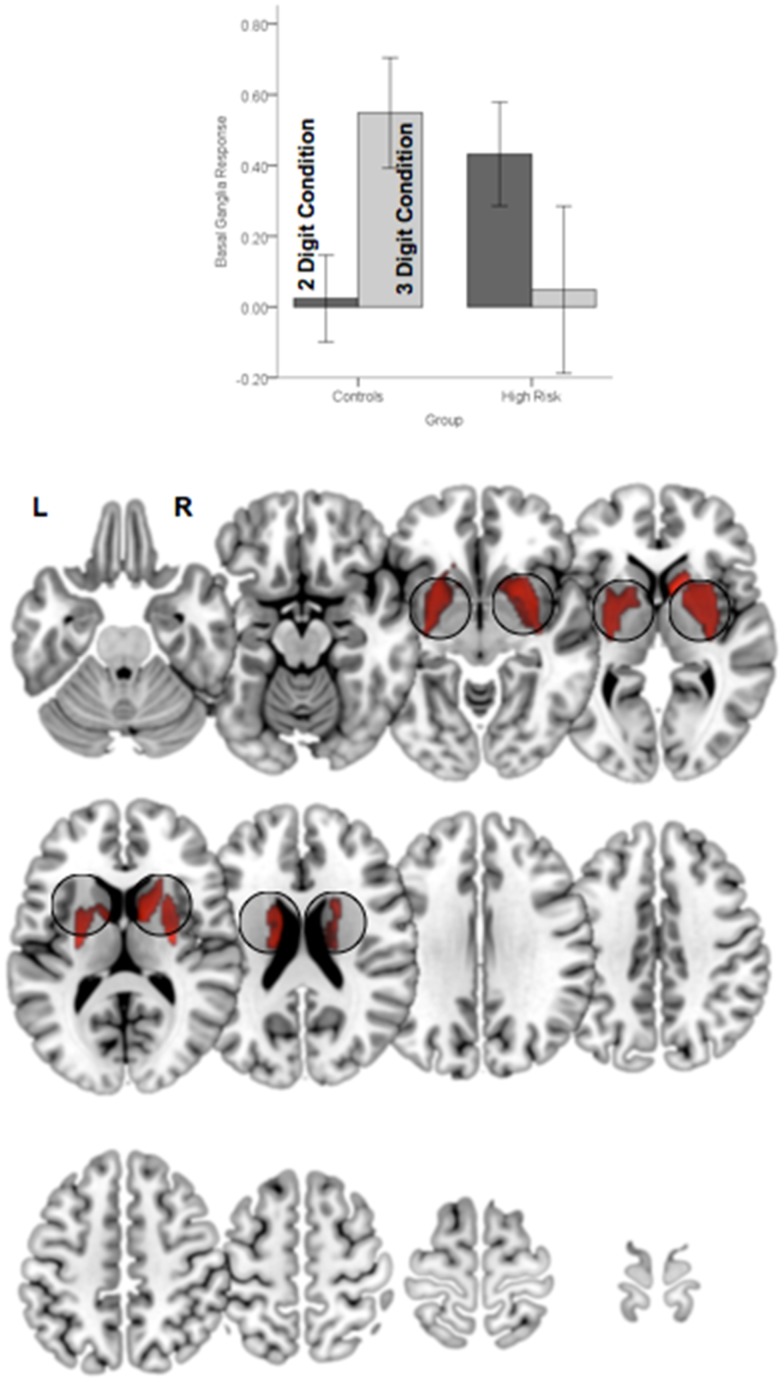
**Significant clusters (*p* < 0.05, cluster level) under the group × attention demand interaction term are rendered on an ascending mosaic of axial views**. As seen in the graph, the interaction was driven by increased engagement with demand in controls, but decreased engagement in high-risk subjects. By implication, the BG, a core region in the executive attention circuit, appears to “turn off” in risk subjects with an increase in attention-related demand.

In addition to the main effect of group a significant Group × Demand interaction in the basal ganglia (Figure 6). As seen in the accompanying graph of the modeled responses, the interaction resulted from an increase in BG engagement with increases in load with a corresponding decrease in engagement in HGR. These activation results suggest that genetic risk confers an imbalance in the patterns of *relative specialization* of attention-related function in adolescence, in particular with diminished engagement on executive regions of the network including the dPFC and the BG, but aberrantly increased reliance on the parietal cortex. The DCM results provide a notable complement for these activation-based analyses by demonstrating the effects of genetic risk in adolescence on the *functional integration* of information across regional networks for attention.

### DCM analyses of fMRI data (differences in functional integration)

**Figure 7:**
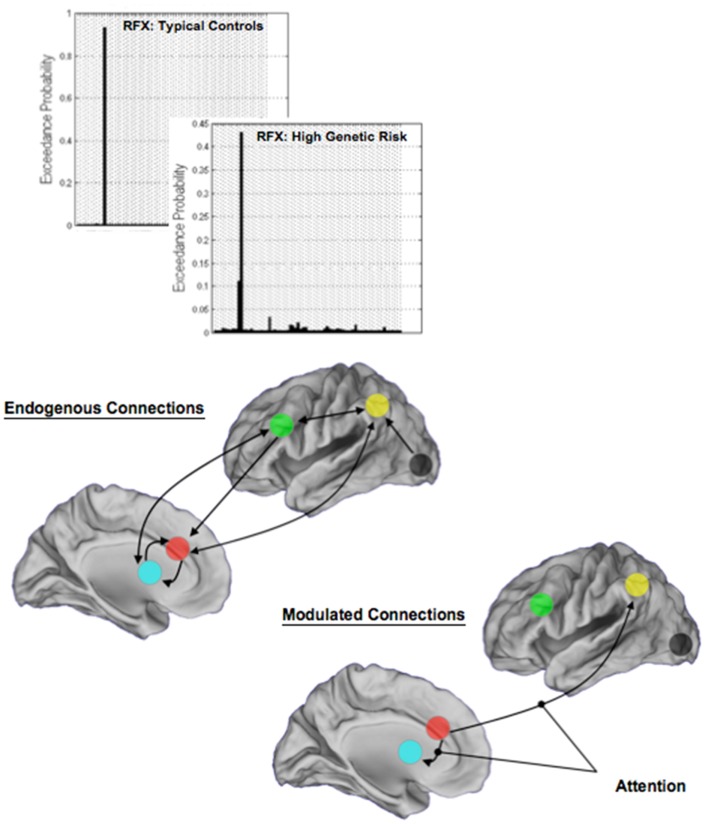
**The results of BMS are depicted in each of the TC and the HGR groups, and resulted in the identical model with the highest evidence**. Model evidence is more heterogeneous in HGR than TC (see Discussion), evidence of intrinsic heterogeneity in this group. The endogenous connectivity of the winning model and the modulation of connections by attention are shown. Note that the primary modulated connections both originated in the dACC, terminating in the BG and the parietal cortex, respectively. The results of modulation suggest that dACC efferents may be particularly relevant in the implementation of attention in the modeled network of regions.

Random-effects analyses and BMS revealed a single winning model in each of TC and HGR. Figure 7 depicts model structure (specifically the pathways modulated by attention) and the observed exceedance probabilities for each of the TC and HGR groups. Notably, these results suggest that the likeliest generative models of the data did not differ across groups, with attention modulating the dACC efferents to the BG and the Parietal cortex. This convergence of model structure implies that any differences in effective connectivity between TC and HGR were to be expected in the parameter estimates of endogenous coupling, or contextual modulation of that coupling by attention (32).

**Figure 8:**
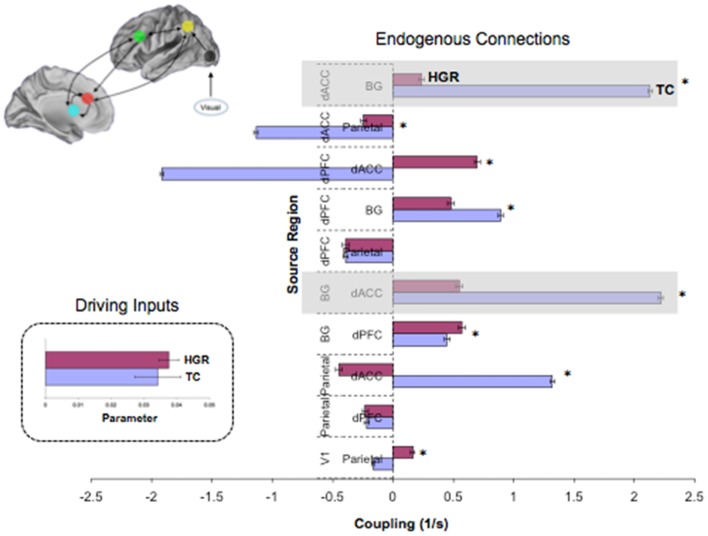
**Endogenous connections and driving inputs in TC and HGR**. Bayesian parameter averages over all subjects in the winning model (Figure 7 reproduced here) are depicted for each connection and for each of TC and HGR (± posterior standard errors). For the graph depicting endogenous connections, the source regions are vertically labeled and the target regions are horizontally labeled. TC and HGR reveal a mixture of positive and negative coupling across the modeled network. The shaded areas draw attention to highly significant hypo-connectivity in the bilateral dACC–BG sub-circuit in HGR subjects. The negative/inhibitory dPFC coupling in TC may reflect competitive inhibition between frontal regions most associated with cognitive control, and is absent in HGR. The inset depicts driving inputs to the primary visual cortex (not different between TC and HGR). Despite model evidence suggestive of identical model structures, these data suggest that HGR are characterized by disordered effective connectivity in the modeled sustained attention network.

**Figure 9:**
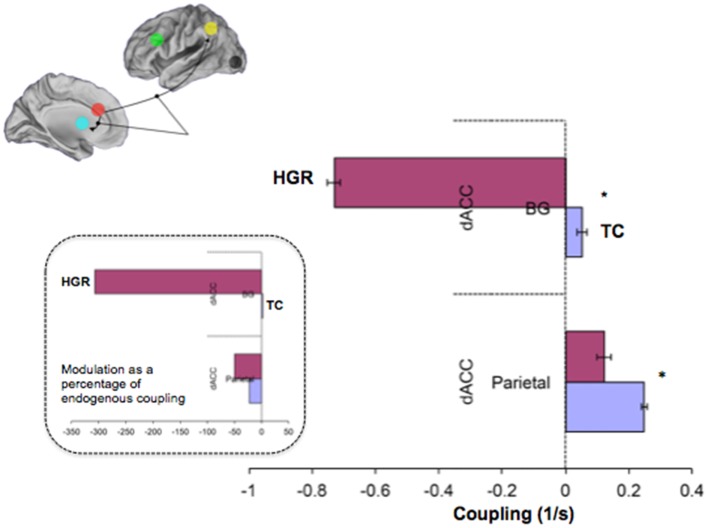
**Contextually modulated connections in the winning model in TC and HGR**. Bayesian parameter averages over all subjects for the winning model (Figure 7 reproduced here) are depicted for each modulated connection (± posterior standard errors). The source regions are vertically labeled and the target regions are horizontally labeled. For both dACC efferent pathways, attention has different patterns of modulation in HGR. The degree of positive modulation (excitatory) on the dACC–Parietal pathway is reduced in HGR, suggesting that the gain in connection strength is lower when implementing attention processing. Moreover, the dACC–BG pathway in HGR is significantly inhibited during attention processing. The inset depicts contextual modulation expressed as a percentage of the endogenous coupling values (Figure 8). This turning down may reflect critical sub-network disengagement in HGR during attention processing and may encode particular vulnerability for impaired attention, cognition, and control that has been documented in HGR groups.

To test for group differences we used the Bayesian parameter average over subjects within each group. This is appropriate because the best model was the same for both groups and therefore a comparison of the group-specific Bayesian parameter averages is unbiased by differences in Bayesian selection. This procedure provides posterior densities over the effective connectivity parameters for both groups, enabling one to estimate the difference between group means and posterior confidence in those differences (shown in terms of a posterior standard error in the figures). Group differences significant at a corrected level of *p* < 0.05, Bonferroni corrected (constituting, *p* < 0.003 for each of the 13 tests) are indicated (*). These *P* values were based upon the posterior differences in group-specific Bayesian parameter averages – and their significance can be visualized in terms of posterior standard errors in the Figures 8 and 9 below.

Figure 8 depicts observed estimates of endogenous coupling for each of the pairwise connections modeled across the endogenous network. As seen, the results provide an admixture of excitatory and inhibitory coupling across network pairs across the task. The most notable and symmetric finding was the bi-directional hypo-connectivity in the dACC ↔ BG pathway observed in HGR compared to TC (matched shaded insets). Notably, relative to TC, in HGR virtually every dACC efferent pathway was characterized by hypo-connectivity, suggesting convergence with hypothesis on the dysfunctional role of the dACC in schizophrenia and mood disorders. In addition, we also observed a difference between TC and HGR on dPFC ↔ dACC and the dACC ↔ Parietal pathways, with TC characterized by inhibitory coupling but HGR characterized by excitatory coupling (former) and decreased inhibitory coupling (latter).

We also observed notably differences in the attention-related contextual modulation of the efferent pathways from the dACC to the BG and the parietal lobe (Figure 9). In both cases, HGR were characterized by attention-related dysmodulation, albeit differing in character. Firstly, during attention epochs the dACC ↔ BG pathway was inhibited in HGR but increased in TC. Secondly, the dACC ↔ Parietal pathway was increase during attention in both groups, but the degree of modulation was reduced in HGR. In the remainder of the paper, we discuss the import of these results in inferring the role of genetic risk on brain networks for attention, and the interpretation of the relationships between the analyses of relative specialization differences (activation) and functional integration differences (effective connectivity). We also reflect on the import role of network analyses of fMRI data in inferring accurate profiles of psychiatric risk in brain networks.

## Discussion

Assessing activation and effective connectivity differences between TC and HGR revealed striking differences in (a) the regional brain responses and interactive effects of attention demand, and (b) patterns of estimated endogenous and contextual effective connectivity between specific sub-networks, particularly related to dACC efferents. Activation analyses revealed an imbalance in regional brain function in HGR: the degree of dPFC engagement was reduced, with an apparent shifting in the relative degree of engagement to the parietal cortex. Furthermore, the BG in TC was responsive to variations in attention load, but disengaged in HGR. These activation-derived imbalances in regional recruitment in HGR suggest a relative shift away from relying on the dPFC and the BG core regions of the executive attention network (37, 67, 68), and toward regions such as the parietal lobe that may be more associated with spatial attention and orientation (69). These activation-based analyses provide a degree of convergence with fMRI patterns observed in adult patients with frank phenotypes of psychosis or mood disorders. For example, forebrain areas in schizophrenia appear hypo-active during conscious and rapid (as opposed to deliberative) processing tasks that engage attention (40, 70, 71). Moreover, in stimulus-response integration tasks with significant attentional demand, regional profiles of engagement are shifted in schizophrenia toward the parietal cortex (72). Similarly, adult bipolar patients are characterized by decreased basal ganglia activity during sustained attention and thalamus during a sustained attention task (73). Given the unique and integrative role of regions such as the dPFC and the basal ganglia in corticostriatal loops subserving attention and memory (74), this disengagement may reflect the role of genetic risk in creating a latent functional deficit in adolescence that alters the relative specialization of function of attention-related regions. This latent deficit is an important vulnerability marker of predisposition to disorders of psychosis or mood (75–77).

Whereas the observed activation-related in HGR appear to foreshadow adult studies in schizophrenia and bipolar disorder, evidence of disordered effective connectivity in HGR constitutes an entirely novel line of inquiry into the functional network biology attention networks and their relationship to risk (29). In general, applications of effective connectivity analyses of fMRI data in adult schizophrenia or bipolar patients has been fruitful in revealing disordered connectivity in tasks of learning (78), sentence completion tasks (79), emotion processing (80), and working memory (81). Perhaps the closest predecessor related to the current set of results is recent work investigating working memory related disordered effective connectivity in young individuals in the prodromal state for schizophrenia (35). In these subjects, reduced frontal–parietal connectivity during working memory (and intermediate between TC and schizophrenia patients) and the implied reduction in functional integration within these critical brain circuits may be predictive of the eventual transition to psychosis. We note that the prodromal state is itself a unique risk-state, constituting an advanced stage of (non-specific) clinical symptoms, and therefore distinct from the HGR group assessed herein. Nevertheless a significant proportion of HGR subjects are likely to transition toward frank phenotypes by way of prodromal symptoms. In this clinical/sub-clinical context, we highlight two points of plausible convergence. Firstly, reduced dACC ↔ BG endogenous connectivity in HGR may reflect a latent dyscoupling in the dormant risk-state that may impair the scaling up of cortical networks to implement higher order tasks. Given the important role of this sub-network in tasks as diverse as memory, attention, motor and cognitive control, and skill learning (82–86), it is likely that a connectivity deficit in this sub-circuit will foreshadow likely deficits in a slew of psychological domains. Indeed, it is unsurprising that in general large neuropsychological assessments of HGR indicate widespread impairments in neurocognitive domains, most of which rely in some form on attention processing (21, 23, 87, 88).

Disordered contextual modulation of dACC ↔ BG and dACC ↔ Parietal efferent pathways provides a parallel and likewise intriguing aspect of network-related dysfunction, particularly as assessments of contextual modulation provide a highly unique contribution of DCM to the study of brain network dynamics and systems theory (33, 89). In this regard, reduced (positive) modulation of the dACC ↔ Parietal pathway and strong inhibition of the dACC ↔ BG pathway in HGR are suggestive of differences of attention-related implementation in the same network. Given that these parameters represent an increase or decrease in connection strength as a function of the implemented task, the inhibition of the dACC ↔ BG pathway indicates the disengagement of this interaction in response to attention processing. As this disengagement is contrary to the expected excitation in TC, it suggests that in addition to being hypo-connected, the dACC ↔ BG sub-network is also “turned down” during attention processing. This turning down (and the disordered response to load) suggests that frontal–striatal network function is sub-optimal in the risk-state. This is consistent with the relationship between dopamine and frontal–striatal function (90), the developmental tuning of the dopamine response, and the relevance of frontal–striatal dopamine dysfunction for schizophrenia and risk for schizophrenia (91).

### Relative specialization and functional integration in HGR

Both the more conventional assessment of regional activation strength and the more advanced analysis of effective connectivity revealed impaired cortical–striatal signals in HGR. Both of them, however, provide unique insight. Activation-based approaches with a general linear model framework do not explicit distinguish between network and/or task constituents (e.g., endogenous connections, modulation by task, sensory inputs) and from the perspective of system’s theory, these approaches are slightly incomplete (89). Thus, the observed disordered activations in HGR are neutral in revealing network-based dysfunction underlying genetic risk and provide more general assessments of differences in the relative engagement of brain regions. We also note that activation-based approaches did not identify HGR-TC differences in regions such as the dACC, perhaps reflecting a limitation in classical statistical approaches to fMRI.

By comparison, DCM is limited by *a priori* assumptions in the assessed network and the structure of the model space. It nevertheless has proven to be more sensitive in identifying abnormal biological signatures in risk-groups, where activation analyses were not. For example, using DCM we recently documented disordered cortical–limbic endogenous connectivity and contextual modulation during an emotional appraisal task in children of schizophrenia parents (34). Notably, this finding emerged despite widespread overlap in activation networks across risk and control groups. DCM thus proved to be highly sensitive in uncovering emergent impairments in functional brain organization, not apparent in regional brain activation patterns or behavioral. It has hence repeatedly been proposed that effective connectivity is an important aspect of research on high-risk samples, not only because of its more realistic interactional model but also because of its reliance on Bayesian statistics (38, 92). By contrast, the absence or restriction of significant regional effects in classical statistics may partly be related to its premises of minimizing the type I error (while Bayesian statistics rely on the highest posterior probability) (28, 32).

### Limitations and prospectus

We conclude with a brief note on the limitations of the study, and a brief note on the prospective role of fMRI in high-risk research. The present study is limited by the relatively small sample size, though this limitation is slightly offset by the robustness of the results, particularly the effective connectivity analyses. Moreover, we acknowledge that HGR is a heterogeneous group, which may explain the more heterogeneous pattern of model evidence for HGR (Figure 7) compared to TC. This heterogeneity is well known and has been characterized before with MRI (25, 36, 93). Moreover, even though children of schizophrenia and bipolar patients are not distinct in terms of attention impairment (assessed with neuropsychological measures) (14), it is plausible that implementation of attention in brain networks may differ. We acknowledge that these differences are not knowable in the current analyses on account of sample size limitations. Also, in assessing effective connectivity, in this first approach, we did not explicitly model effects of attention load (providing a point of asymmetry with the activation-based analyses), though we are currently augmenting our analyses to investigate these effects.

The study of adolescents at genetic risk for schizophrenia or bipolar disorder offers opportunities and challenges. As indicated previously, HGR constitute a unique risk group, distinct from prodromal or clinical high-risk samples (94–97). Studying HGR in the medication naïve state can provide interesting insights into the intersection of genetic risk and abnormal neurodevelopment (98, 99). By focusing on a *profile of cumulative genetic risk*, rather than on *individual genes*, such approaches are important given the polygenic and non-specific genetic bases of psychiatric disorders (100, 101). However, the emergence of frank phenotypes (typically in early adulthood) is mediated by a host of unknown and uncontrolled factors (102), and neurobiological signatures in HGR may be non-specific and carry uncertain predictive value. Nevertheless, we suggest that a focus on critical domains such as sustained attention, and understanding of brain network dysfunction underlying these domains in HGR may provide a particularly fruitful path forward in understanding how genetically mediated vulnerability is encoded in disordered brain network interactions.

## Author Contributions

Vaibhav A. Diwadkar designed and supervised the project, data acquisition and all analyses and interpretation. Neil Bakshi conducted DCM analysis. Gita Gupta conducted activation bases analysis. Patrick Pruitt and Richard White assisted in data collection and analyses. Simon B. Eickhoff collaborated with Vaibhav A. Diwadkar in design, analyses, and interpretation.

## Conflict of Interest Statement

The authors declare that the research was conducted in the absence of any commercial or financial relationships that could be construed as a potential conflict of interest.
